# Vagus nerve preservation and double-flap technique in laparoscopic proximal gastrectomy

**DOI:** 10.3389/fsurg.2025.1656058

**Published:** 2025-11-21

**Authors:** Ke-Kang Sun, Xiao-Jun Shen, Peng Hua, Yi-Fan Zhang, Yong-You Wu

**Affiliations:** 1 Department of Gastrointestinal Surgery, Affiliated Kunshan Hospital to Jiangsu University, Suzhou, Jiangsu, China; 2 Department of Gastrointestinal Surgery, The Second Affiliated Hospital of Soochow University, Suzhou, Jiangsu, China

**Keywords:** proximal gastrectomy, laparoscopy, double-flap, vagus nerve, gastroesophageal reflux disease

## Abstract

**Objectives:**

Laparoscopic proximal gastrectomy has been a common treatment strategy for proximal early gastric cancer. The conventional double-flap technique (DFT), also called Kamikawa method, does not require vagus nerve preservation, which is precisely crucial to maintain quality of life and prevent postoperative reflux esophagitis.

**Methods:**

A single-center retrospective analysis was conducted on 37 gastric cancer patients undergoing laparoscopic proximal gastrectomy with vagus nerve preservation and double-flap technique. The hepatic and celiac branches were both preserved. A seromuscular double-flap was created through the auxiliary incision, and the anastomosis between the oesophagus and the remnant stomach was performed under laparoscopy.

**Results:**

2.7% of the patients suffered from slight anastomotic stricture but subsequently recovered after conservative treatment. No patients experienced anastomotic bleeding or leakage. No food residue and GERD (Los Angeles classification grade B or higher) were observed in any patients 6 months later. Ultrasonography showed that the gallbladder contractile function was normal in all patients.

**Conclusion:**

Although long-term follow-up and a larger number of patients are required to evaluate the functional outcomes, our technique provides a minimally invasive surgical option for proximal early gastric cancer, especially in the prevention of postoperative reflux esophagitis.

## Introduction

The Japanese gastric cancer treatment guidelines (JGCG) defined standard gastrectomy as the adequate stomach resection and D2 lymph node dissection (1). Due to a low incidence of lymph node metastasis and excellent long-term survival in early gastric cancer (EGC), function-preserving gastrectomy (FPG) was started for EGC to reduce surgical invasiveness and address the postoperative quality of life (QOL) (2–4). The primary aim of FPG involved the preservation of the autonomic nerves as well as the maintenance of the physiological gastrointestinal hormonal secretion. Thus, apart from reducing the extent of gastrectomy, the surgeons aimed to preserve the pylorus and the perigastric vagus nerve during the surgical procedure (5). Actualy, pylorus-preserving gastrectomy (PPG) and proximal gastrectomy (PG), which were thought to be the ideal methods to fulfill the three elements of FPG, were generally performed in EGC according to limited indications.

The prevalence of proximal EGC has been increasing continuously during the past two decades in Western and Asian countries (6, 7). Accompanying this trend, PG was labeled as FPG and preferred over total gastrectomy (TG) as it could mitigate the nutritional deterioration and weight loss associated with the latter (8, 9). It was expected that PG would preserve the remaining functioning of the remnant distal stomach, including the pyloric ring function that prevented biliary reflux as well as a lower rate of dumping syndrome (10, 11). However, patients who undergo PG might also suffer from esophageal reflux, which could lead to poor QOL (12–14). JGCG proposed three types of reconstructions, namely, esophagogastrostomy (EG), double-tract reconstruction (DTR), and jejunal interposition (JI) (1). EG is simplified by its requirement of a single anastomosis and conforms to physiological structure, and it is usually performed with another anti-reflux procedure, such as gastric tube (15), side overlap (16) or double-flap reconstruction (DFR) (17). The perigastric vagus nerve mainly refers to the hepatic branches and the celiac branches. The preservation of the hepatic branches could prevente postoperative gallstones formation and facilitated gastric motility (18). The celiac branches were related to the motility of the duodenum and the proximal jejunum, as well as the regulation of gastrointestinal hormone secretion (19). In the present study, laparoscopic PG with VNP and double-flap technique (DFT) was successfully performed in 37 patients, representing a novel technique for proximal EGC. The preliminary results and technical aspects of the surgical technique were discussed.

## Materials and methods

### Patients

From March 2020 to May 2022, 37 patients underwent laparoscopic PG with VNP and DFT. Our research strictly followed the guidelines of the Helsinki Declaration, and received ethical approval from The Second Affiliated Hospital of Soochow University. In addition, all study subjects provided informed consents prior to the initiation of the study. Indications for this surgery included the tumor being located in the upper third of the stomach without esophageal invasion, the depth of tumor invasion confined to T1, no lymph node involvement, and lesions that could not be treated by endoscopic mucosal resection or local resection. Patients underwent upper gastrointestinal angiography before discharge. The six-month follow-up visit involved a postoperative interview regarding gastroesophageal reflux disease (GERD Q scoring systems). All patients received endoscopic and ultrasonic examination 6 months later to determine GERD (Los Angeles classification grade B or higher) and gallbladder contraction function. Demographic and clinicopathological characteristics were summarized using descriptive analysis (mean ± SD).

### Surgical techniques

The patients received general anesthesia and were positioned supine. The surgeon and assistant stayed on each side of the patient, while the scopist stayed in the middle. After creating a pneumoperitoneum at the umbilicus, we inserted a 2D laparoscope into one of the four (5 or 12 mm) ports on each side of the patient's upper abdomen.

**Figure 1:**
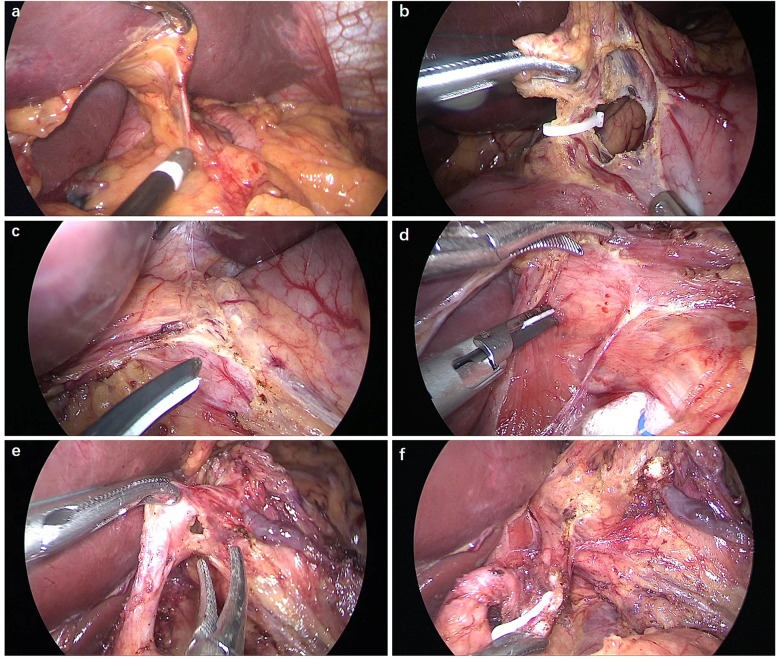
Preservation of hepatic branches and celiac branches. **(a)** The hepatogastric ligament was separated below the hepatic division to retain the hepatic branches. **(b)** The second branch of the right gastric artery was dissected. **(c)** The front of the abdominal oesophagus was exposed. **(d)** The posterior vagus nerve trunk was excised between the oesophagus and diaphragmatic crus. **(e)** The left gastric artery was separated just distal to the celiac division junction and left gastric artery to preserve the celiac branches. **(f)** The lymph node was dissected whilst preserving the celiac branches.

Firstly, the omentum was dissected from the mesocolon around the transition zone of lymph node station 4d whilst preserving the right gastroepiploic vessels. Any posterior adhesion of the stomach was dissected. Next, the retroperitoneum was proximally dissected away along the spleen till the left gastroepiploic vessels were recognized and they were then divided using hemoclips (station 4sb). The short gastric arteries were dissected close to the upper spleen pole (station 4sa). Lymph nodes stations 11d and 10 were not routinely dissected. Then, the gastric fundus was isolated by separating the gastrodiaphragmatic ligament. To prevent injury to the hepatic branches that emerge from the anterior trunk of the vagus nerve, they were located after a midline incision was made to fenestrate the gastrohepatic ligament (Figure 1a). The anterior gastric branches were identified and dissected by following the vagus nerve's hepatic branches to their proximal sides. Afterwards, Laparoscopic proximal gastrectomy was accomplished using vagus nerve preservation and the double-flap approach in patients who had their stomachs preserved to a two-thirds level (Figure 1b). The surrounding tissue of the abdominal oesophagus was shifted to the actinal side, exposing the front of the abdominal oesophagus (Figure 1c). Dissecting the retroperitoneum involves opening the anterior crus of the right diaphragm and taping the trunk of the posterior vagus nerve by exfoliating the oesophagus. When the posterior vagus nerve was traced, the posterior gastric branches could be located and dissected (Figure 1d). When the left gastric artery was close to the celiac ganglia, it was contacted by celiac branches that had branched off from the posterior trunk. They proceeded to split and cut the left gastric vein. The distal celiac branches were used to cut and divide the left gastric artery (Figure 1e). Retracting the posterior trunk of the vagus nerve toward the surgeon allowed for the dissection of lymph node along the left gastric artery (Figure 1f). The posterior gastric artery was severed and dissection continued through the spleen (station 11p) and lymph node stations 8a and 9. A 60-mm Endo-Gia endoscopic linear stapler was used to transect the exposed oesophagus. A 5-cm incision was made in the patient's upper abdominal region, through which the stomach was removed. The surgeon observed the lesions and transected the stomach with a linear stapler whilst maintaining an adequate surgical margin and retaining two-thirds of the stomach.

**Figure 2:**
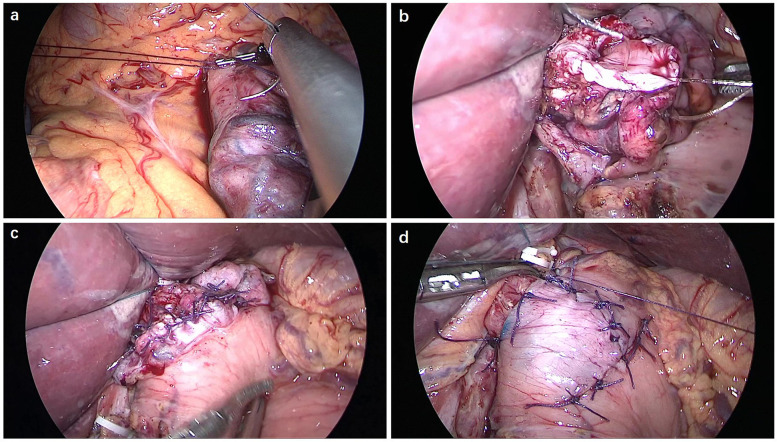
Double-flap technique of the oesophagus and gastric remnant. **(a)** Three-point sutures were made between the posterior side of the oesophagus and the upper edge of the seromuscular flap on the remnant stomach. **(b)** The posterior wall was anastomosed using a V-Loc continuous suture. **(c)** The anterior wall was anastomosed using a layer-to-layer suture. **(d)** The anastomotic site was covered with the seromuscular double-flap.

By making a further incision around 1–2 cm from the proximal resection stump, a seromuscular double-flap (2.5 cm × 3.5 cm) was created on the front surface of the remaining stomach. The connective tissue between the muscle and the mucosa was cut under proper tension. The gastric mucosa was exposed for anastomosis, keeping a 1 cm gap from the lower flap end. Anastomotic stricture was prevented by keeping this gap slightly greater than the width of the oesophagus. At the flap's upper edge, the posterior side of the oesophagus was attached to the remaining stomach using three-point sutures (Figure 2a). Hand sewing method was used to anastomosize the oesophagus to the stomach remnant using a V-Loc suture (Figure 2b). For the posterior wall, a continuous single-layer suture was used between all layers of the oesophagus and gastric mucosa. The anterior wall was sutured using a layer-to-layer technique, with one continuous suture connecting the oesophagus and stomach mucosa, and another interrupted suture connecting the stomach seromuscular and oesophageal muscular layers (Figure 2c). The Y-shaped flap was then secured in place along the midline to encompass the biggest feasible region of the anastomosis (Figure 2d).

## Results

The present study included 11 female and 26 male patients with a median age of 68 years and a median BMI of 23.2. Cancer invaded the mucosa in 17, and submucosa in 20 patients. A median of 270 min (245–320 min) and 70 mL of blood was lost during surgery (range of 40–130 mL). All patients were provided a liquid diet on the second day after operation. The x-rays taken one week after operation. One patient suffered from slight anastomotic stricture but subsequently recovered after conservative treatment. The contrast media passed smoothly through the anastomosis into the duodenum, resulting in no marked regurgitation into the oesophagus in all the patients. Patients did not suffer from postoperative anastomotic bleeding or leakage, wound-related issues, pancreatic fistulae, or luminal bleeding. Lung infection occurred in 2 patients but was resolved with antibiotic treatment. The mean postsurgical hospitalization was 9.6 ± 4.1 days. Symptoms of gastroesophageal reflux were noted in 3 patients and these symptoms were relieved by antacids 6 months later. All patients received endoscopic and ultrasonic examination. No food residue and GERD (Los Angeles classification grade B or higher) were observed in any patients. B-mode ultrasonography showed that the volume of the gallbladder was large in 2 patients and the contractile function was normal in all patients (Table 1).

**Table 1:** Clinicopathologic characteristics and surgical outcomes of 37 patients who underwent LPG with VNP and DFT.

Variable	Value
Age (years; median)	68 (35–81)
Sex	
Male/female	26/11
BMI (kg/m^2^; median)	23.2 (20.8–28.3)
Tumor size (cm; mean ± SD)	2.0 ± 1.5
Depth of invasion	
pT1a/ pT1b	17/20
pN stage	
N0/N1/N2/N3	35/2/0/0
Resected lymph nodes (number; median)	31.1 (23–45)
Time of surgery (min; mean ± SD)	270 ± 45
Conversion to open laparotomy	0
Hospital-stay post-operation (day; mean ± SD)	9.6 ± 4.1
Complication	
Anastomosis leakage	0
Anastomosis stricture	1 (2.7%)
Anastomosis bleeding	0
Pancreatitis/Pancreatic fistula	0
Intra-abdominal bleeding	0
Wound problem	0
Lung infection	2 (5.4%)
Gallbladder contractile dysfunction	0
Food residue and reflux esophagitis	0
Symptoms of gastroesophageal reflux	3 (8.1%)
Mortality	0

## Discussion

The increase in the incidence rate of proximal EGC has attracted attention to study the long-term prognosis as well as improved postoperative QOL. Compared with TG, PG has been found to result in reduced supplemental feeding, decreased incidences of vomiting and diarrhea, as well as improved ability to maintain weight loss (8). EG requires a single anastomosis and conforms to a physiological structure. However, the major concern for EG is reflux esophagitis, which adversely affects the post-operative QOL. Two reasons can explain this result, i.e., the resection of the cardia and the injury of the vagus nerve. Thus, ideal reconstruction methods were investigated to prevent reflux esophagitis. Kamikawa described an anti-reflux procedure based on EG, which was also called DFR (17). A multicenter retrospective study has provided evidence supporting the feasibility and utility of this method (20). The critical parts of this reconstruction involved the creation of the seromuscular double flap and the implanted length of the esophagus, which affected esophageal reflux symptoms as the flap would provide a one-way valve function. Although DFR was effective for treating gastroesophageal reflux, it could not resolve the anastomoses. Therefore, a routine intraoperative gastroscopy was performed during the operation to check the size and patency of the anastomosis (21). Furthermore, it was found that the diameter of the esophagus <18 mm was an independent risk factor for postoperative anastomotic stenosis with DFR (22, 23). In our series, we did not perform this reconstruction for patients with the diameter of the esophagus <2 cm. Intraoperative gastroscopy was not routinely performed, and none of the patients developed anastomotic stricture. Preserving as much remnant stomach as possible is another important factor in maintaining postoperative QOL. Nomura et al. (24) found that EGC patients benefited from half gastrectomy rather than the typical two-thirds gastrectomy in laparoscopic distal gastrectomy (DG). Under the premise of ensuring sufficient margin, it was recommended to preserve two-thirds of the stomach in our surgical procedure.

On the other hand, the vagus nerve plays an important role in the functional integrality of abdominal organs. The perigastric vagus nerve mainly refers to the hepatic branches and the celiac branches. It has been previously reported that the hepatic branches correlated with the function of the liver and biliary system, facilitated bile excretion, and prevented postoperative gallstone formation, which might reduce the incidence of cholecystolithiasis (18, 25, 26). Moreover, the hepatic branches have been shown to facilitate gastric motility post-gastrectomy (19). The celiac branches were known to associate with the motility of the upper gastrointestinal tract in dogs (27). Similar results were seen in humans, and the celiac branches were related to the motility of the duodenum and the proximal jejunum (19). Furthermore, the celiac branches were also related to the regulation of gastrointestinal hormone secretion. Takiguchi et al. (28) reported that the celiac branches were essential in postprandial ghrelin reduction, which was considered necessary for maintaining homeostasis and controlling energy balance. However, the vagus nerve was usually resected during radical gastrectomy, which caused post-gastrectomy syndromes, such as diarrhea, delayed gastric emptying, and gallstone. It has been reported that preserving the hepatic branches could prevent postoperative gallstone formation and maintain gastric motility after laparoscopic DG and PPG (18, 29). Nunobe et al. (30) reported that preserving the celiac branches decreased the delayed emptying as compared to the previous results of PPG. A randomized controlled trial revealed that preservation of the vagus nerve could significantly reduce diarrhea and appetite loss at 12 months postoperation compared with conventional distal gastrectomy (31). Although some studies about vagus nerve preservation on gastric motility and diarrhea were controversial (32), these contradictory results could be explained by possible unnoticed injuries to the vagus nerve during surgical procedure. Since PG preserves the distal stomach and pylorus, preservation of the vagus nerve is theoretically more meaningful. A limited number of studies were designed to investigate the value of vagus nerve preserving in PG with lower esophageal sphincter preserved (33–36). However, under the premise of ensuring sufficient margin, the lower esophageal sphincter resection is inevitable in most cases.

Considering the role of gastric motility and cardia sphincter in anti-reflux, we performed LPG with vagus nerve preservation and DFR in the present study. The hepatic and celiac branches were routinely preserved in our surgical procedure. In fact, the hepatic branches were very thin in most cases, which were usually resected inadvertently during surgery. Compared with open surgery, the laparoscopic view allows magnified visualization, which made it easier to perform this delicate operation with accuracy. We located the hepatic branches in laparoscopic view. After fenestration of the gastrohepatic ligament via an upper midline incision, the hepatic branches bifurcating from the anterior trunk of the vagus nerve were identified and were fastened to protect them from damage. After dissecting the retroperitoneum, we opened the anterior surface of the right crus of the diaphragm and taped the trunk of the posterior vagus nerve by exfoliating the esophagus. The celiac branches splitting from the posterior trunk approached the left gastric artery as it neared the celiac ganglion. The left gastric artery was clipped and divided at the point distal to the celiac branches. Retraction of the posterior trunk of the vagus nerve toward the surgeon enabled the lymph node dissection along the left gastric artery. Although there were many variations in the relationship between the celiac branches and the left gastric artery (37), we consider that preserving the celiac branches did not affect the dissection of the stations 7 lymph node in most cases.

In the present study, we successfully performed LPG with VNP and DFT in 37 patients. All patients received endoscopic and ultrasonic examination. No food residue and reflux esophagitis occurred in any of the patients. B-mode ultrasonography showed that the gallbladder contractile function was normal in all patients. The present study has some limitations. First, this was a retrospective study with a small sample size at a single institution. Second, quality of life was not evaluated in these patients because it was not fully followed-up using a validated questionnaire. Third, the comparison of outcomes did not include other reconstructions, including esophagogastrostomy, jejunal interposition, and double-tract reconstruction, after LPG. A randomized clinical trial with equivalent background characteristics among the reconstructions after LPG is required to further analyze the advantages of our method.

## Conclusion

Although long-term follow up and a larger number of patients are required to evaluate functional outcomes, our new technique provides a minimally invasive surgical option for proximal EGC, especially in the cardiac area. A randomized clinical trial with equivalent background characteristics among the reconstructions after LPG is required to further analyze the advantages of our method.

## Data Availability

The raw data supporting the conclusions of this article will be made available by the authors, without undue reservation.

## References

[1] Japanese Gastric Cancer Association. Japanese Gastric cancer treatment guidelines 2014 (ver. 4). Gastric Cancer. (2017) 20:1–19. 10.1007/s10120-016-0622-4 PMC5215069 27342689

[2] Nomura E Okajima K. Function-preserving gastrectomy for gastric cancer in Japan. World J Gastroenterol. (2016) 22(26):5888–95. 10.3748/wjg.v22.i26.5888 27468183 PMC4948261

[3] Nunobe S Hiki N. Function-preserving surgery for gastric cancer: current status and future perspectives. Transl Gastroenterol Hepatol. (2017) 2:77. 10.21037/tgh.2017.09.07 29034350 PMC5639020

[4] Saito T Kurokawa Y Takiguchi S Mori M Doki Y. Current status of function-preserving surgery for gastric cancer. World J Gastroenterol. (2014) 20(46):17297–304. 10.3748/wjg.v20.i46.17297 25516640 PMC4265587

[5] Nomura E Isozaki H Fujii K Toyoda M Niki M Sako S Postoperative evaluation of function-preserving gastrectomy for early gastric cancer. Hepatogastroenterology. (2003) 50(54):2246–50. 14696509

[6] Ahn HS Lee HJ Yoo MW Jeong SH Park DJ Kim HH Changes in clinicopathological features and survival after gastrectomy for gastric cancer over a 20-year period. Br J Surg. (2011) 98(2):255–60. 10.1002/bjs.7310 21082693

[7] Dassen AE Lemmens VE van de Poll-Franse LV Creemers GJ Brenninkmeijer SJ Lips DJ Trends in incidence, treatment and survival of gastric adenocarcinoma between 1990 and 2007: a population-based study in The Netherlands. Eur J Cancer. (2010) 46(6):1101–10. 10.1016/j.ejca.2010.02.013 20219351

[8] Takiguchi N Takahashi M Ikeda M Inagawa S Ueda S Nobuoka T Long-term quality-of-life comparison of total gastrectomy and proximal gastrectomy by postgastrectomy syndrome assessment scale (PGSAS-45): a nationwide multi-institutional study. Gastric Cancer. (2015) 18(2):407–16. 10.1007/s10120-014-0377-8 24801198

[9] Masuzawa T Takiguchi S Hirao M Imamura H Kimura Y Fujita J Comparison of perioperative and long-term outcomes of total and proximal gastrectomy for early gastric cancer: a multi-institutional retrospective study. World J Surg. (2014) 38(5):1100–6. 10.1007/s00268-013-2370-5 24310733

[10] Katai H Sano T Fukagawa T Shinohara H Sasako M. Prospective study of proximal gastrectomy for early gastric cancer in the upper third of the stomach. Br J Surg. (2003) 90(7):850–3. 10.1002/bjs.4106 12854112

[11] Shiraishi N Adachi Y Kitano S Kakisako K Inomata M Yasuda K. Clinical outcome of proximal versus total gastrectomy for proximal gastric cancer. World J Surg. (2002) 26(9):1150–4. 10.1007/s00268-002-6369-6 12209245

[12] Yoo CH Sohn BH Han WK Pae WK. Long-term results of proximal and total gastrectomy for adenocarcinoma of the upper third of the stomach. Cancer Res Treat. (2004) 36(1):50–5. 10.4143/crt.2004.36.1.50 20396565 PMC2855111

[13] An JY Youn HG Choi MG Noh JH Sohn TS Kim S. The difficult choice between total and proximal gastrectomy in proximal early gastric cancer. Am J Surg. (2008) 196(4):587–91. 10.1016/j.amjsurg.2007.09.040 18519129

[14] Rosa F Quero G Fiorillo C Bissolati M Cipollari C Rausei S Total vs proximal gastrectomy for adenocarcinoma of the upper third of the stomach: a propensity-score-matched analysis of a multicenter western experience (on behalf of the Italian research group for gastric cancer-GIRCG). Gastric Cancer. (2018) 21(5):845–52. 10.1007/s10120-018-0804-3 29423892

[15] Adachi Y Inoue T Hagino Y Shiraishi N Shimoda K Kitano S. Surgical results of proximal gastrectomy for early-stage gastric cancer: jejunal interposition and gastric tube reconstruction. Gastric Cancer. (1999) 2(1):40–5. 10.1007/s101200050019 11957069

[16] Yamashita Y Yamamoto A Tamamori Y Yoshii M Nishiguchi Y. Side overlap esophagogastrostomy to prevent reflux after proximal gastrectomy. Gastric Cancer. (2017) 20(4):728–35. 10.1007/s10120-016-0674-5 27942874

[17] Kuroda S Nishizaki M Kikuchi S Noma K Tanabe S Kagawa S Double-Flap technique as an antireflux procedure in esophagogastrostomy after proximal gastrectomy. J Am Coll Surg. (2016) 223(2):e7–13. 10.1016/j.jamcollsurg.2016.04.041 27157920

[18] Wang CJ Kong SH Park JH Choi JH Park SH Zhu CC Preservation of hepatic branch of the vagus nerve reduces the risk of gallstone formation after gastrectomy. Gastric Cancer. (2021) 24(1):232–44. 10.1007/s10120-020-01106-z 32705445

[19] Kong SH Kim SM Kim DG Park KH Suh YS Kim TH Intraoperative neurophysiologic testing of the perigastric Vagus nerve branches to evaluate viability and signals along nerve pathways during gastrectomy. J Gastric Cancer. (2019) 19(1):49–61. 10.5230/jgc.2019.19.e2 30944758 PMC6441774

[20] Kuroda S Choda Y Otsuka S Ueyama S Tanaka N Muraoka A Multicenter retrospective study to evaluate the efficacy and safety of the double-flap technique as antireflux esophagogastrostomy after proximal gastrectomy (rD-FLAP study). Ann Gastroenterol Surg. (2018) 3(1):96–103. 10.1002/ags3.12216 30697614 PMC6345660

[21] Muraoka A Kobayashi M Kokudo Y. Laparoscopy-Assisted proximal gastrectomy with the hinged double flap method. World J Surg. (2016) 40(10):2419–24. 10.1007/s00268-016-3510-5 27094564

[22] Shoji Y Nunobe S Ida S Kumagai K Ohashi M Sano T Surgical outcomes and risk assessment for anastomotic complications after laparoscopic proximal gastrectomy with double-flap technique for upper-third gastric cancer. Gastric Cancer. (2019) 22(5):1036–43. 10.1007/s10120-019-00940-0 30838469

[23] Hayami M Hiki N Nunobe S Mine S Ohashi M Kumagai K Clinical outcomes and evaluation of laparoscopic proximal gastrectomy with double-flap technique for early gastric cancer in the upper third of the stomach. Ann Surg Oncol. (2017) 24(6):1635–42. 10.1245/s10434-017-5782-x 28130623

[24] Nomura E Lee SW Bouras G Tokuhara T Hayashi M Hiramatsu M Functional outcomes according to the size of the gastric remnant and type of reconstruction following laparoscopic distal gastrectomy for gastric cancer. Gastric Cancer. (2011) 14(3):279–84. 10.1007/s10120-011-0046-0 21519869

[25] Kojima K Yamada H Inokuchi M Kawano T Sugihara K. Functional evaluation after vagus-nerve-sparing laparoscopically assisted distal gastrectomy. Surg Endosc. (2008) 22(9):2003–8. 10.1007/s00464-008-0016-8 18594924

[26] Uyama I Sakurai Y Komori Y Nakamura Y Syoji M Tonomura S Laparoscopic gastrectomy with preservation of the vagus nerve accompanied by lymph node dissection for early gastric carcinoma. J Am Coll Surg. (2005) 200(1):140–5. 10.1016/j.jamcollsurg.2004.07.035 15631934

[27] Shahidullah M Kennedy TL Parks TG. The vagus, the duodenal brake, and gastric emptying. Gut. (1975) 16(5):331–6. 10.1136/gut.16.5.331 1140630 PMC1411071

[28] Takiguchi S Hiura Y Takahashi T Kurokawa Y Yamasaki M Nakajima K Preservation of the celiac branch of the vagus nerve during laparoscopy-assisted distal gastrectomy: impact on postprandial changes in ghrelin secretion. World J Surg. (2013) 37(9):2172–9. 10.1007/s00268-013-2078-6 23645130

[29] Tomita R Fujisaki S Koshinaga T Kusafuka T. Clinical assessments in patients ten years after pylorus-preserving gastrectomy with or without preserving both pyloric and hepatic branches of the vagal nerve for early gastric cancer. Hepatogastroenterology. (2010) 57(101):984–8. 21033264

[30] Nunobe S Hiki N Fukunaga T Tokunaga M Ohyama S Seto Y Laparoscopy-assisted pylorus-preserving gastrectomy: preservation of vagus nerve and infrapyloric blood flow induces less stasis. World J Surg. (2007) 31(12):2335–240. 10.1007/s00268-007-9262-5 17952497

[31] Kim SM Cho J Kang D Oh SJ Kim AR Sohn TS A randomized controlled trial of Vagus nerve-preserving distal gastrectomy versus conventional distal gastrectomy for postoperative quality of life in early stage gastric cancer patients. Ann Surg. (2016) 263(6):1079–84. 10.1097/SLA.0000000000001565 26727095

[32] Furukawa H Ohashi M Honda M Kumagai K Nunobe S Sano T Preservation of the celiac branch of the vagal nerve for pylorus-preserving gastrectomy: is it meaningful? Gastric Cancer. (2018) 21(3):516–23. 10.1007/s10120-017-0776-8 29127549

[33] Matsumoto H Murakami H Kubota H Higashida M Nakamura M Hirai T. Clinical outcome of lower esophageal sphincter- and vagus-nerve-preserving partial cardiectomy for early gastric cancer of the subcardia. Gastric Cancer. (2015) 18(3):669–74. 10.1007/s10120-014-0389-4 24906556

[34] Hirai T Matsumoto H Iki K Hirabayashi Y Kawabe Y Ikeda M Lower esophageal sphincter- and vagus-preserving proximal partial gastrectomy for early cancer of the gastric cardia. Surg Today. (2006) 36(10):874–8. 10.1007/s00595-006-3265-y 16998680

[35] Tomita R. Surgical techniques to prevent reflux esophagitis in proximal gastrectomy reconstructed by esophagogastrostomy with preservation of the lower esophageal sphincter, pyloric and celiac branches of the vagal nerve, and reconstruction of the new his angle for early proximal gastric cancer. Surg Today. (2016) 46(7):827–34. 10.1007/s00595-015-1269-1 26671623

[36] Matsui H Igarashi N Itano O Koyama Y Miyakita M. Laparoscopic function-preserving surgery for early gastric cancer in the upper third of the stomach: vagus-sparing proximal gastrectomy with side-to-side esophagogastric-tube anastomosis. Tokai J Exp Clin Med. (2007) 32(4):109–14. 21318948

[37] Ando S Tsuji H. Surgical technique of vagus nerve-preserving gastrectomy with D2 lymphadenectomy for gastric cancer. ANZ J Surg. (2008) 78(3):172–6. 10.1111/j.1445-2197.2007.04396.x 18269482

